# Development of Rifampicin Eye Drops for the Treatment of Exudative Age-Related Macular Degeneration

**DOI:** 10.3390/ph18050655

**Published:** 2025-04-29

**Authors:** Valory Anne S. Vailoces, Andrew J. Tolentino, Jose Fernando Arevalo, Ron A. Adelman, Robert Bhisitkul, Diana V. Do, Quan Dong Nguyen, Michael J. Tolentino, Masaki Tanito, Hiroaki Serizawa

**Affiliations:** 1 School of Medicine & Health Sciences, George Washington University, Washington, DC 20052, USA; valory_vailoces@gwu.edu; 2 Department of Biology, University of California Berkeley, Berkeley, CA 94720, USA; atolent@berkeley.edu; 3 Retina Division, Wilmer Eye Institute, Department of Ophthalmology at Johns Hopkins Bayview Medical Center, Johns Hopkins University, Baltimore, MD 21287, USA; arevalojf@jhmi.edu; 4 Department of Ophthalmology and Visual Science, School of Medicine, Yale University, New Haven, CT 06510, USA; ron.adelman@yale.edu; 5 Department of Ophthalmology, Mayo Clinic, Jacksonville, FL 32224, USA; 6 Department of Ophthalmology, University of California San Francisco, San Francisco, CA 94158, USA; robert.bhisitkul@ucsf.edu; 7 Department of Ophthalmology, Byers Eye Institute, School of Medicine, Stanford University, Palo Alto, CA 94303, USA; dianado@stanford.edu (D.V.D.); ndquan@stanford.edu (Q.D.N.); 8 Department of Ophthalmology, School of Medicine, University of Central Florida, Orlando, FL 32827, USA; mtolent88@me.com; 9 Department of Ophthalmology, Orlando College of Osteopathic Medicine, Winter Garden, FL 34787, USA; 10 Department of Ophthalmology, Shimane University Faculty of Medicine, Shimane 693-8501, Japan; mtanito@med.shimane-u.ac.jp; 11 AMD Therapeutics LLC, Palo Alto, CA 94306, USA; 12 NeoVascularX, Inc., Palo Alto, CA 94306, USA

**Keywords:** macular degeneration, rifampicin, rifamycin, anti-angiogenesis, VEGF, eye drops, neovascularization

## Abstract

**Background/Objectives**: Exudative age-related macular degeneration (AMD) is a disease of choroidal neovascularization that causes blindness. Current treatments to preserve vision in this prevalent and blinding condition are repeat intraocular injections of anti-vascular endothelial growth factor medicines for a patient’s lifetime to preserve and prevent vision loss leading to blindness. Rifampicin, a small-molecule antibiotic, has previously been reported to exhibit anti-angiogenic properties and a topical safety profile that is well-tolerated. Based on this evidence, we investigated the feasibility of formulating rifamycin as an ophthalmic drop capable of delivering therapeutic concentrations to the posterior segment of the eye. **Methods**: Inhibition of neovascularization by administration of rifampicin was analyzed in the rat oxygen-induced retinopathy (OIR) and mouse laser-induced choroidal neovascularization (CNV) models. Pharmacokinetic (PK) studies were conducted in mice, rats, and rabbits by dosing various formulations containing rifampicin, and the compound was quantified by LC/MS analysis. **Results**: Results from dose escalation studies in the mouse laser-induced CNV model suggested the minimum effective dose of rifampicin required for inhibiting neovascularization in subretinal tissues to be 0.7 mg/kg, which is substantially lower than the 20 mg/kg dosage approved for infectious disease treatments. The previous studies did not report the minimum effective dose in the anti-angiogenesis effects. The effective area under the concentration-time curve (AUC) in the sub-retina was evaluated as 0.27 h·ng/mg. In rabbits, rifampicin was delivered to the sub-retina by a single topical application of various formulations in a dose-dependent manner. The topical application of the formulations containing 1% rifampicin, which was well-tolerated in clinical trials previously reported for ocular trachoma, achieved subretinal delivery approximately 2–32 times greater than the effective AUC. Plasma exposure of the compound by the topical application was evaluated to range approximately 0.5–10 ng/mL. **Conclusions**: Rifampicin was delivered to the sub-retina in rabbits with an efficiency greater than the effective dose required for inhibiting neovascularization. Limited amounts of plasma exposure by the topical application were detected. These results suggested the therapeutic potential of the rifampicin formulations for the topical treatment of exudative macular degeneration.

## 1. Introduction

Subretinal and retinal neovascularization in ophthalmologic diseases such as diabetic retinopathy, age-related macular degeneration, retinal vein occlusion, and retinopathy of prematurity is a leading cause of irreversible visual loss and blindness. Currently, 20 million Americans and 196 million people globally are affected by age-related macular degeneration (AMD). Fleckenstein et al. reported that AMD is predicted to have a prevalence of 288 million people worldwide by 2040 [1]. AMD begins with the development of subretinal deposits called drusen within the macula, the area of the retina that discerns central vision. The prolonged presence of drusen can lead to advanced AMD characterized by choroidal neovascularization (exudative AMD) or geographic atrophy (dry AMD), which is the permanent loss of photoreceptors, retina cells, and retinal pigment epithelium [2,3].

The current standard treatment for exudative AMD is anti-vascular endothelial growth factor (VEGF) therapy administered intravitreally [1]. Past treatment options that are no longer widely used for exudative AMD include thermal laser therapy, photodynamic therapy, and surgical excision of neovascular lesions [4]. These treatment options involve the inhibition or removal of neovascularization. Therefore, they do not reverse the permanent photoreceptor destruction. Similarly, diabetic retinopathy and hemiretinal vein occlusion are managed with anti-VEGF therapy and laser therapy, with intravitreal steroids as second-line treatment [5,6]. Anti-VEGF therapy, while effective, requires patients to frequently visit their healthcare provider for repeat eye injections, which cause discomfort, anxiety, and a risk of endophthalmitis.

Intravitreal anti-VEGF therapies are antibodies, fusion protein traps, and fAb fragments that are not conducive to sustained delivery, systemic, oral, or topical routes of administration. Because retinal conditions require prolonged and frequent administration, the ideal method of delivery would be eyedrops. There are currently no topical products for exudative AMD approved by the FDA. Several companies are working on an extended-release to reduce the number of injections, but a topical treatment would provide a more patient-friendly alternative to monthly eye injections. Potential limitations of the topical treatment include variability in dosage and patient adherence [7]. However, these limitations can be mitigated by achieving efficient drug delivery to the posterior segment of the eye.

Topical eyedrops, while easier to use by patients, face multiple developmental challenges, such as anatomic barriers that limit drug absorption, permeability, and efficacy. As a result, eyedrops must be formulated at higher drug concentrations, which can potentially lead to anterior segment side effects [8].

Steroid eyedrop formulations, specifically glucocorticoids, are common for ophthalmologic conditions due to their anti-inflammatory, anti-angiogenic, and immunosuppressive effects. Commonly used topical steroids include fluorometholone acetate, loteprednol etabonate, prednisolone acetate, dexamethasone phosphate, and hydrocortisone acetate [9]. For acute inflammation, steroid treatments typically yield expected outcomes. However, these treatments are not suitable for managing chronic inflammation or neovascularization associated with chronic conditions, such as those observed in exudative AMD.

Given the established efficacy of anti-VEGF drugs, tyrosine kinase inhibitors (TKIs) have been targeted for the development of eyedrops to treat exudative AMD. These eye drops include reformulated versions of TKIs previously approved for cancer treatments, such as regorafenib [10]. Moreover, TKIs suitable for inhibiting neovascularization in the back of the eye have been identified from small-molecule libraries using both in vitro and in vivo assays. Compounds such as acrizanib and PAN-90806 have been formulated into eyedrops [11,12]. However, clinical trials for these products have been unsuccessful. The significant cytotoxicity of TKIs has led to adverse effects in the anterior segment of the eye, and insufficient amounts of these TKIs reach the posterior segment. Although increasing the TKI concentrations or viscosity in eyedrops is generally expected to enhance their delivery to the posterior segment, this needs to be balanced with safety considerations. This suggests that a limited number of TKIs will be qualified to be developed as eyedrops for exudative AMD.

The eyedrop formulation of regorafenib was showing promising results in laser-induced CNV model studies in monkeys. However, it did not pass phase 2 efficacy trials, primarily due to insufficient delivery to the back of the eye [10]. Additionally, notable differences in drug delivery efficiency across multiple species were identified, attributed mainly to the varying thickness of scleral tissue and eyeball size across different species. For example, monkeys have thicker sclera than rabbits, necessitating a more efficient delivery method for monkeys. Human eyes, being larger than monkey eyes, require an even more efficient delivery method.

Pazopanib was the first TKI to be reformulated into an eyedrop and tested in exudative AMD [13,14]. It was also evaluated as an oral medication [15]. In the eyedrop trial, only patients with the high-risk genotype (CFH Y402H TT) responded with a reduction of macular edema [14,16]. Conversely, in the oral trial, patients who received the rescue therapy had the high-risk genotype, and nonrescued patients experienced improvements in visual acuity, central retinal lesion thickness, and central retinal thickness. Subsequently, phase 2 trials enrolling 510 AMD patients for 7 different dosing groups were conducted to evaluate the safety and efficacy of the eyedrop in combination with ranibizumab. However, no benefit beyond the effect of ranibizumab was detected [16,17]. The development of the pazopanib eyedrop was stopped because of the trial failure. However, these trials and their outcomes have informed future development and trial designs. The trials were designed with daily topical dosing frequencies of three and four times, suggesting that reducing the frequency of dosing could enhance consistency in dosage and patient adherence. Additionally, specific trial designs might need to focus on evaluating the injection interval of anti-VEGF therapies to further explore the potential of pazopanib eyedrops.

There are several other eyedrops currently in development that have potential as exudative AMD therapy. EXN407 is a serine-arginine protein kinase 1 (SRPK1) inhibitor that acts on VEGF alternative splicing. The EXN407 Phase Ib/IIa trial data demonstrated safety and biological activity in the treatment of diabetic retinopathy and diabetic macular edema [18]. Fenofibrate, a cholesterol drug, has been correlated to decreased AMD and has also reduced the need for laser treatment in diabetic retinopathy [19,20]. Oculis has developed a topical dexamethasone ophthalmic suspension, which has demonstrated a reduction in central macular thickness in patients with diabetic macular edema with visual improvement in eyes with lower baseline vision [21].

**Figure 1:**
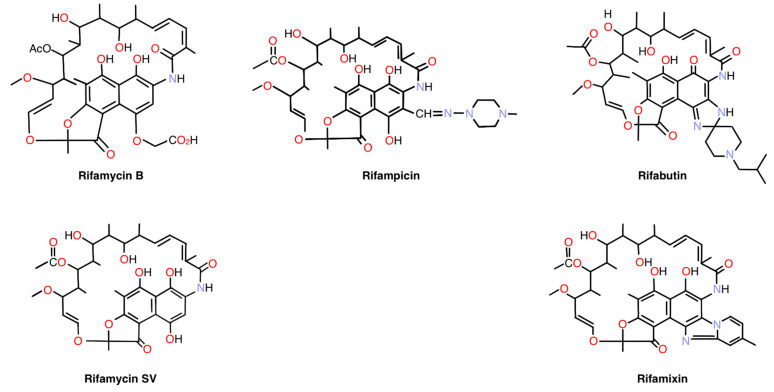
Chemical structures of Rifamycin B, Rifamycin SV, Rifampicin, Rifabutin, and Rifamixin.

Rifampicin as an eyedrop formulation is a novel concept for neovascular diseases of the eye. Rifampicin is a chemical modification of rifamycins (chemical structures shown in Figure 1), metabolites of *Streptomyces mediterranei* [22]. Rifamycins inhibit DNA-dependent RNA synthesis via high-affinity binding to prokaryotic RNA polymerase [23,24,25,26]. Rifampicin is a component of the combination therapy against *Mycobacterium tuberculosis* and is effective against Gram-positive prosthetic joint and valve infections that often involve biofilms. Ocular tuberculosis is an extrapulmonary manifestation of tuberculosis (TB) and is managed the same as pulmonary tuberculosis [27].

Rifampicin’s anti-angiogenetic effects were found in treatments for patients with both pulmonary tuberculosis and hepatocellular carcinomas [28]. Oral administrations of rifampicin for pulmonary tuberculosis showed a significant decrease in tumor progression and cancer biomarker levels in these patients, suggesting that the compound is potent in inhibiting neovascularization in humans [28,29]. Furthermore, oral administration of rifampicin inhibited neovascularization induced by human sarcoma that was implanted into the dorsal air sac of a mouse [29]. It was shown to inhibit tube formation and cell proliferation of human umbilical vein endothelial cells (HUVEC) in vitro and retinal neovascularization in the retinopathy of prematurity oxygen-induced mouse model [30].

Rifampicin, while used extensively in tuberculosis patients, has several known associated side effects, including nausea, vomiting, diarrhea, loss of appetite, and a change in urine color. A concerning side effect of the typical six-month course for tuberculosis is hepatotoxicity, which has been shown in up to 28% of patients taking rifampicin [31]. This serious side effect of systemically administered rifampicin precludes it from clinical use as a treatment for exudative macular degeneration or proliferative diabetic retinopathy, which are currently being treated with local intraocular injections of anti-VEGF drugs. Intraocular injections are systemically safe. In addition, those on intermittent or long-term rifampicin therapy may experience immunoallergic effects such as cutaneous, gastrointestinal, or flu-like syndromes, hemolytic anemia, shock, or kidney injury [32]. Rifampicin’s toxicity may be due in part to drug-drug interactions since the compound is usually administered as part of a multi-drug regimen. It is also prone to drug-drug interactions due to CYP3A4 induction, affecting the metabolism of over half of all clinically relevant drugs [33,34,35]. The pharmacokinetics of rifampicin can also be affected by gastrointestinal or liver xenobiotics, especially in those with predisposed select polymorphisms [36]. Given these adverse effects of rifampicin, to utilize the compound for exudative AMD, the development of topical formulations seems to be a reasonable approach. If concentrations of the compound are localized in ocular tissues, the adverse effects will be avoided.

Darougar et al. reported that thrice daily topical application of 1% rifampicin ointment, administered for up to seven weeks, was generally well tolerated in clinical use [37,38]. Five to ten percent of the patients enrolled in the trials experienced discomfort and tearing. Improvements in the rifampicin ointment formulation, including micronized particle sizes, could further optimize the safety profiles. The local ocular safety profiles of rifampicin further encouraged us to develop topical formulations to treat exudative AMD.

In this paper, we demonstrate the highly specific inhibition of neovascularization by rifampicin in the mouse laser-induced CNV model, which has also been utilized in the preclinical development of anti-VEGF drugs such as ranibizumab and aflibercept. Our topical formulations efficiently delivered rifampicin to the posterior segment of the eye. We found that an order of magnitude greater than the effective dose was delivered to the posterior segment in rabbits and monkeys. Given the potent anti-angiogenic effects and the ability of these topical formulations to achieve effective concentrations at the posterior pole, this approach represents a promising strategy to extend the intervals between anti-VEGF injections or potentially eliminate the need for intravitreal injections.

## 2. Results

### 2.1. Topical Application of Rifampicin Reaches the Retina

Table 1 shows the quantification of rifampicin, which was extracted from the retinal tissues that were topically dosed with 0.25% rifampicin. Rifampicin was delivered to the retina in a dose-dependent manner.

**Figure 2:**
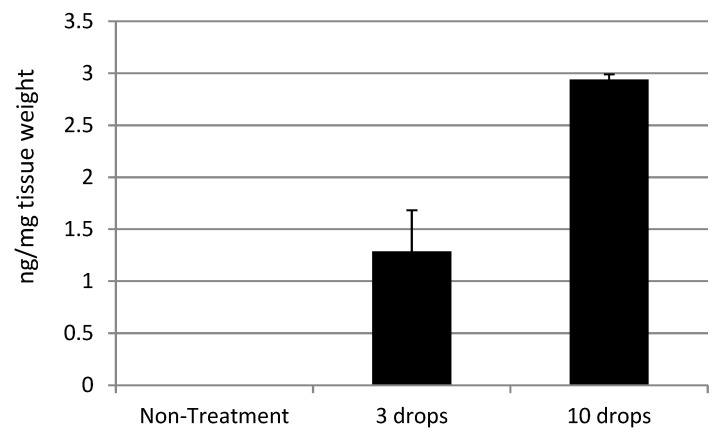
Amount of rifampicin delivered to rat retina by application of topical eye drops (0.25% rifampicin). (Average n = 4).

Retinal delivery of rifampicin by the topical eyedrop formulation shown in Figure 2 was over 100 times more efficient than that of dexamethasone. Among other drugs listed in Table 1.

### 2.2. Pharmacokinetic Studies Using a 0.25% Rifampicin Eye Drop Formulation

**Figure 3:**
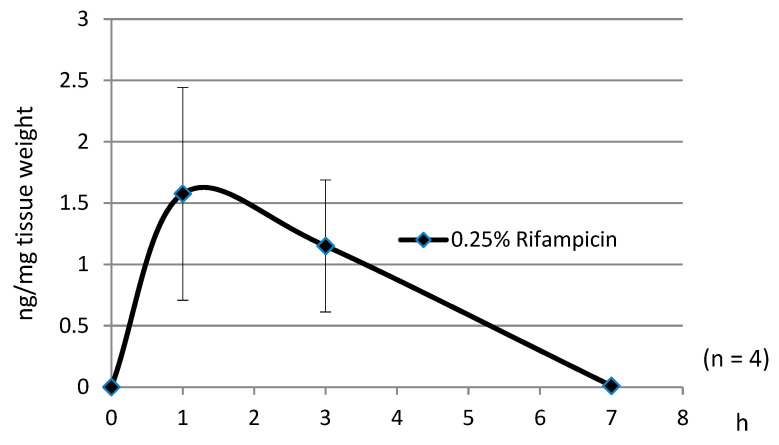
Amount of rifampicin delivered to rat retina by a single topical application of 0.25% rifampicin formulation. T_max_: about 1 h, T_1/2_: about 3–4 h. (Average n = 4).

Figure 3 presents the pharmacokinetic results obtained from studies using 15 μL of a 0.25% rifampicin eye drop formulation. Rifampicin was delivered to the rat retina by a single topical application, achieving T_max_ of approximately 1 h and T_1/2_ of 3–4 h. These pharmacokinetic profiles support the feasibility of topical treatment at an appropriate frequency.

### 2.3. Preclinical Efficacy

**Figure 4:**
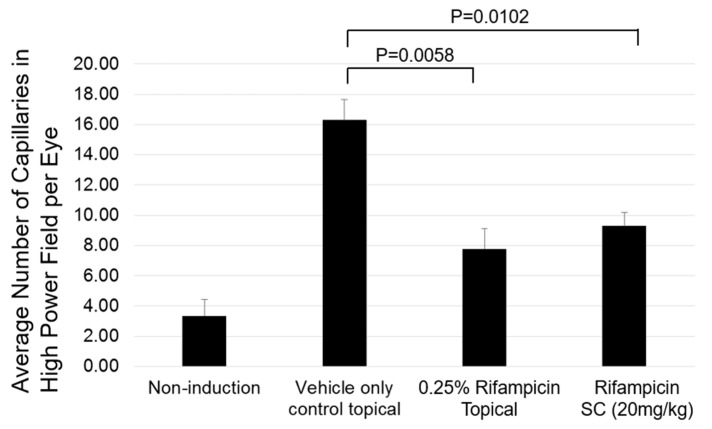
Neovascularization in the retina of oxygen-induced retinopathy rat models was quantified by the detection of capillaries in histological sections.

Data shown in Figure 4 provide the results of a preclinical efficacy test performed on oxygen-induced retinopathy rat models using a 0.25% rifampicin eye drop formulation. Figure 4 shows the quantification of capillaries detected in the histology sections.

Representative images of the histology sections are shown in Figure S1A–H (Please see Supplementary Materials). The retina treated with the control of only a vehicle showed an increased number of small new capillaries on the retinal surface (Figure S1A,B). The retina treated with the AMD 101 topical eye drop formulation showed a small proliferation focus of new capillaries on the retinal surface, but the number of new capillaries was fewer than that in the control group (Figure S1C,D). The retina treated with rifampicin subcutaneous (SC) injection showed a small proliferation focus of new capillaries on the retinal surface, but the number of new capillaries is fewer than that in the control group (Figure S1E,F). The retina, in which retinopathy was not induced, showed a few small vessel cross-sections but did not show any capillaries (Figure S1G,H).

### 2.4. Determination of Effective Dose of Rifampicin in Mouse Laser-Induced CNV Models

**Figure 5:**
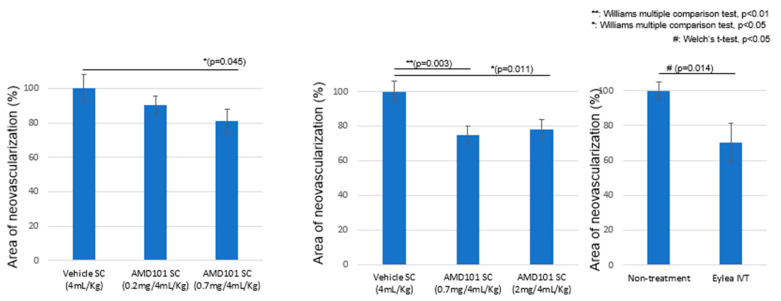
Inhibition of neovascularization by SC injection of rifampicin in mouse laser-induced CNV model.

Data obtained in Figure 5 provides the results of mouse laser-induced CNV models after exposure to vehicle-only SC, rifampicin SC (0.2 mg/kg), and rifampicin SC (0.7 mg/kg).

These results suggest that an SC injection with a dosage of 0.7 mg/kg significantly inhibited neovascularization in the CNV model. An effective systemic dosage of rifampicin to inhibit bacterial infection was previously evaluated at 20 mg/kg. Therefore, the effective dosage to inhibit neovascularization in the sub-retina in the CNV model was approximately 30 times less than that of the bacterial inhibition. The neovascularization inhibition level achieved through SC injections of rifampicin at doses exceeding 0.7 mg/kg was equivalent to that achieved with intravitreal injection of aflibercept.

**Figure 6:**
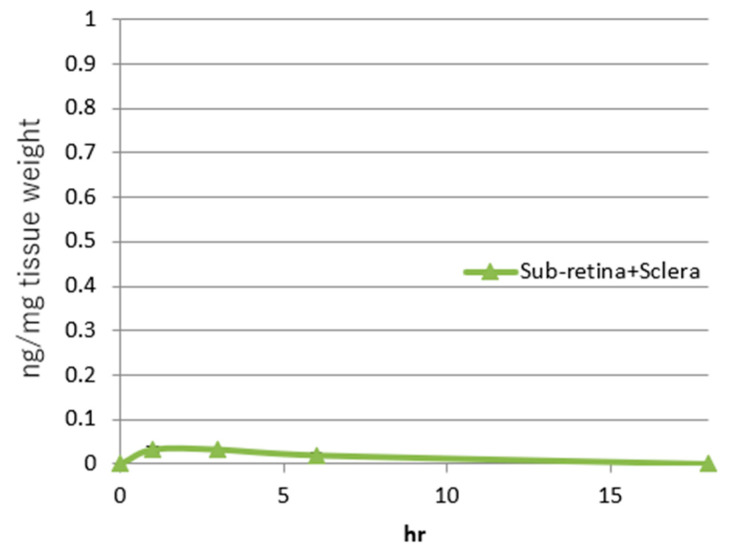
Delivery of rifampicin to the sub-retina/sclera complex of mouse eyes by a single subcutaneous injection (0.7 mg/kg). (Average n = 3).

The average rifampicin delivered to the sclera and sub-retina complex (ng/mg tissue) by a single SC injection (0.7 mg/kg) is shown in Figure 6.

The effective AUC was evaluated as approximately 0.27 (h·ng/mg tissue). At each time point, the standard errors ranged from 0.005334 to 0.006478, and Figure 6 includes the narrow error bars. Therefore, the efficacy of rifampicin in inhibiting neovascularization is expected to be seen when equal to or more than the effective AUC is delivered to the sub-retinal tissue. In separate mouse pharmacokinetic studies by SC injections of rifampicin, delivery ratios between sub-retinal and scleral tissues were evaluated as approximately 7:3, respectively. Therefore, approximately 70% of rifampicin delivered to the sub-retina and sclera complex was evaluated to be delivered to the sub-retina (WO 2023/234423 A1).

### 2.5. Ocular Tissue Delivery of Rifampicin via Oil-Based, Water-Based Suspension, and Water-Solubilized Formulations by Topical Applications

**Figure 7:**
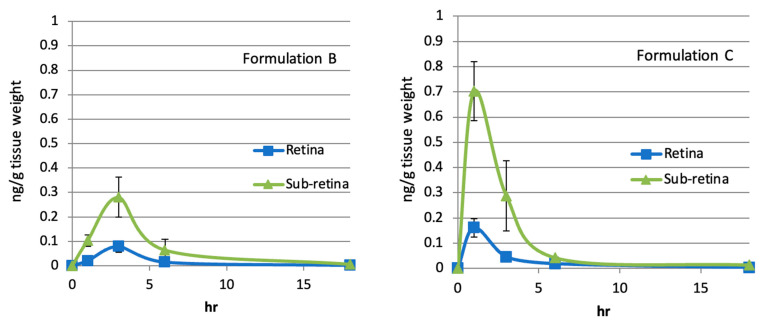

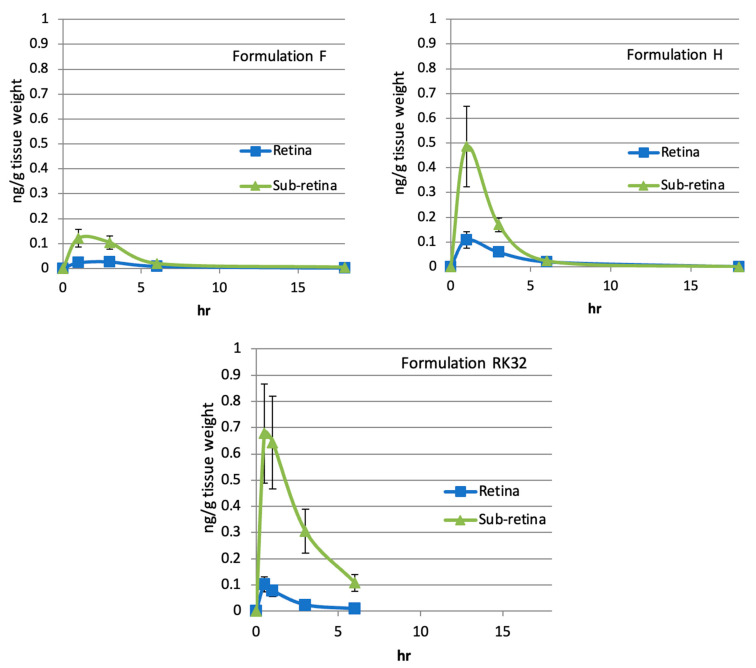
Pharmacokinetic analysis of a single topical application of Formulations B, C, F, H, and RK32. (Average n = 3).

The mouse effective AUC was compared with AUC values connecting time points between 1 h and 18 h, with each average concentration detected in the retina and sub-retina obtained in the rabbit studies using the oil-based formulations (A–F), the water-based suspension formulations (G and H), and the water-solubilized formulation (RK32). These formulations contained 1% rifampicin. AUC data for sub-retina (Table 2) and retina (Table 3) are shown below. Pharmacokinetic analysis of a single topical application of Formulation B, C, F, H, and RK32 is shown in Figure 7.

These data suggested that all the oil-based formulations (A–F), the water-based suspension formulation (G and H), and the water-solubilized formulation (RK32) delivered to the retinal and sub-retinal tissues rifampicin amounts equivalent to or larger than the effective AUC that was required for inhibition of neovascularization in mouse CNV models by topical applications. Similar pharmacokinetic results were obtained in monkeys when an oil-based formulation containing 1% rifampicin was topically applied (Unpublished data). Therefore, when these oil- or water-based formulations containing 1% rifampicin are applied to the eyes of animals or humans at least once daily, neovascularization in sub-retinal or retinal tissues will be effectively inhibited. This inhibition is attributed to the fact that the AUC values achieved by a single topical application of these formulations exceeded the threshold for the therapeutic concentration. Thus, the formulations presented here can be utilized for treating posterior neovascular eye diseases, including AMD and diabetic retinopathy. Consequently, patients will experience increased intervals of repeated intravitreal injections, alleviating the associated treatment burden.

### 2.6. Oil-Based Formulation Plasma Exposure

In the pharmacokinetic studies described in Table 2 and Table 3 and Figure 7, oil- and water-based formulations (A–D and RK32) containing 1% rifampicin were topically applied to the eyes of the rabbits. One hour after the topical application, blood samples were collected from the rabbits, and plasma fractions were prepared from the blood samples to evaluate systemic exposures to rifampicin. Rifampicin in the plasma fractions was quantified by LC/MS (Table 4). The detection limit was 0.25 ng/mL plasma.

Plasma exposures detected by dosing the oil-based formulations (A-D) were significantly smaller than those of the water-based formulation (RK32). Improvements in the oil formulations were evaluated in the plasma exposure. These plasma exposures were approximately or less than a thousandth of that reported by oral dosing for infectious treatments. Therefore, the plasma exposures were highly limited, and topically dosed rifampicin was localized in ocular tissues. The risk associated with potential systemic safety concerns, including hepatotoxicity, was significantly mitigated.

### 2.7. Sub-Retinal and Retinal Delivery of Rifampicin via Oil-Based Formulations

To determine the necessary rifampicin concentration in oil-based formulations for achieving effective AUC in sub-retinal tissues by a single topical application, we evaluated two formulations. Oil-based formulation C, topically dosed at 20 μL per application, contained rifampicin at concentrations of 0.01% and 0.001%. Similarly, oil-based formulation E, also topically dosed at 20 μL, contained rifampicin at concentrations of 0.1% and 0.01%. Rabbits received these formulations topically, and sub-retinal tissues were subsequently extracted at 1, 3, 6, and 18 h post-application. Rifampicin concentrations in these tissues were quantified using LC/MS with a detection limit set at 0.25 ng/g of tissue.

**Table 5:** AUC values were delivered to the sub-retina by a single topical application of Formulation C containing 0.001% and 0.01% rifampicin and Formulation E containing 0.01% and 0.1% rifampicin. (Average n = 3).

	Mouse effective AUC (h·ng/mg tissue)	Sub-retinal AUC by oil-based formulation C 0.01% rifampicin (h·ng/mg tissue)	Sub-retinal AUC by oil-based formulation C 0.001% rifampicin (h·ng/mg tissue)
AUC values	0.27	0.01	0.001
Folds Greater Than Effective AUC	X1	X0.04	X0.004
	Mouse effective AUC (h·ng/mg tissue)	Sub-retinal AUC by oil-based formulation E 0.1% rifampicin (h·ng/mg tissue)	Sub-retinal AUC by oil-based formulation E 0.01% rifampicin (h·ng/mg tissue)
AUC values	0.27	0.27	0.18
Folds Greater Than Effective AUC	X1	X1	X0.7

These data suggest correlations between rifampicin concentrations in the formulations and delivery efficiencies. The approximately 0.01% concentration was required to achieve the effective AUC by the topical application.

## 3. Discussion

This paper’s results confirmed that rifampicin could inhibit neovascularization in the mouse laser-induced CNV model. Chikaraishi et al. previously reported that systemic administration of rifampicin inhibited ischemic neovascularization in a mouse OIR model [30]. Our results align with these previous results. The inhibition levels achieved by rifampicin were equivalent to those achieved by the intravitreal injection of the anti-VEGF drug. Our results confirmed rifampicin’s efficacy in inhibiting neovascularization induced by both ischemia and inflammation.

Furthermore, we demonstrated that topical application of an eyedrop formulation containing rifampicin inhibited neovascularization in the oxygen-induced retinopathy model (Figure 4). Thus, the inhibition of neovascularization by rifampicin was achieved not only by SC injection but also by topical eyedrop application.

Based on the results produced in the CNV model and subsequent pharmacokinetic studies, both the minimum effective dose and AUC were determined. We determined the minimum effective dose of rifampicin for neovascularization inhibition in the CNV model to be approximately 1/30th of the dose approved for treating infections. Originally, rifampicin was discovered and developed for infectious treatments, and specific interactions with prokaryotic RNA polymerase have been elucidated. However, this paper revealed that the specificity of rifampicin’s anti-angiogenic effects is significantly higher than that of its anti-bacterial effects. The high specificity is crucial to developing eye drops to treat exudative AMD.

Additionally, the topical formulations tested delivered a dose to the sub-retinal tissues in rabbits that were about 2–32 times higher than the effective dose (Table 2). Pharmacokinetic studies in monkeys suggested similar delivery efficiencies, indicating potential applicability for delivering the compound to human sub-retinal tissues with comparable efficiency to the intravitreal injection of anti-VEGF drugs (Unpublished data). Plasma exposure levels of rifampicin from the topical application were roughly less than a thousandth of those from the oral dosing approved for infectious treatments, indicating that topical dosing produces highly localized concentrations in ocular tissues (Table 4).

Although oral administration of rifampicin could theoretically treat exudative AMD without systemic side effects such as liver toxicity, the use of topical formulations offers more targeted and efficient delivery to the posterior segment of the eye. Achieving sub-retinal delivery levels equivalent to those by the topical formulations would require oral doses close to those approved for infectious treatments, posing significant risks due to systemic side effects. Therefore, the topical formulations we developed represent promising treatment options for exudative AMD.

It has been revealed that the topical skin application of 2.5% rifampicin ointment inhibited neovascularization in human skin tissues and significantly improved skin redness caused by chronic dermatitis and acne (US patent No. 10,709,702 B2). These findings align with and support the ocular results obtained in the pre-clinical model efficacy and pharmacokinetic data.

The concentration required to achieve statistically significant inhibition of HUVEC tube formation and proliferation in vitro was approximately 30 µM of rifampicin [30]. An actual effective concentration of rifampicin will be lower than this concentration because the compound is likely precipitated or bound with bovine serum under the conditions where HUVECs are cultured in vitro (US Patent Application No. 18/267,715) [30]. Furthermore, the observed concentration closely approximates the peak plasma exposure of rifampicin, which ranges from 9 to 17 µg/mL following a 600 mg intravenous dose and from 4 to 32 µg/mL after a 600 mg oral dose, as reported in the package insert [41]. The former corresponds to an estimated serum concentration of approximately 12–23 µM. In contrast, the peak concentration of rifampicin in the mouse sub-retinal and scleral complex, resulting from a single subcutaneous injection of the effective dose (0.7 mg/kg), was approximately 0.03 ng/mg tissue weight (Figure 5 and Figure 6), which calculates to roughly 0.04 µM. These findings indicate a significant discrepancy between the effective doses evaluated in in vitro HUVEC experiments and those in the mouse CNV model. Beyond the inhibition of growth rates and tube formation in HUVECs, additional mechanisms may contribute to the inhibition of neovascularization by rifampicin.

Khan et al. reported that a daily oral dosage of 300 mg of rifampicin improved visual acuity and macular thickness in patients with Central Serous Chorioretinopathy (CSC) [42]. Furthermore, Khorram reported that Packo et al. identified improvements in CSC with a 600 mg daily oral dose of rifampicin [43]. These studies suggest that rifampicin may improve blood vessel permeability in CSC. Shichiri et al. reported that the addition of rifampicin to in vitro cultures of human dermal microvascular endothelial cells inhibited the expression of genes required for neovascularization, including angiogenic factors such as VEGF and their receptors [29]. This gene inhibition may play a role in improving blood vessel permeability. Results from our pre-clinical model efficacy and pharmacokinetics studies indicate that the concentration of rifampicin delivered to the posterior segment of the eye by oral administration might be comparable to or less than that achieved by the topical formulations, suggesting potential benefits of the topical application in treating CSC.

Darougar et al. reported that thrice daily topical application of 1% rifampicin ointment, administered for up to seven weeks, was well tolerated in clinical use [37,38]. In contrast, our non-GLP tox studies in rabbits revealed no toxicity with twice daily topical application of 1% rifampicin for 3-month dosing (Unpublished data). This suggests that a maximum tolerated concentration of rifampicin could potentially exceed 1%, and higher concentrations might further improve the delivery efficiency to the back of the eye. Data shown in Figure 2 and Table 5 suggested dose dependencies of rifampicin formulations in the delivery to the back of the eye and supported the possibility of the improvement of the delivery efficiency by the higher concentrations. The preliminary safety profiles of rifampicin have mitigated potential concerns about anterior segment toxicity, such as corneal inflammation, that were suggested in the development of Rock inhibitors [44].

To determine conditions that stably solubilize rifampicin in an aqueous phase, we conducted over 5000 high-throughput assays. Through these assays, we elucidated the relationships between rifampicin, various buffers, pH levels, as well as ionic and non-ionic detergents and their concentrations required for solubilization (US Patent Application No. 18/267,715). The insights gained will facilitate the manufacture of water-soluble formulations containing rifampicin. These formulations are designed for efficient delivery of the compound to the posterior segment of the eye, along with oil-based formulations. In this paper, Formulation RK32 demonstrated efficient delivery (Table 2 and Table 3). Oil-based formulation B maintained the stability of rifampicin for over 1.5 years at room temperature (Unpublished data). This suggests that the commercial distribution of the formulation will not require special storage conditions.

The water-soluble formulation RK32 demonstrated approximately an 8-fold higher AUC in the subretinal tissues of rabbits compared to the effective AUC threshold. In contrast, oil-based formulations exhibited delivery efficiencies roughly 10-fold or greater than the effective AUC. Notably, systemic plasma exposures from both oil-based and water-soluble formulations were limited to approximately one-thousandth or less of the exposure observed with oral administration used for infectious disease treatment, although some variation was observed depending on the formulation. While the specific delivery mechanisms underlying the enhanced subretinal targeting of these formulations remain to be elucidated, particle size might not significantly influence delivery efficiency or systemic exposure.

As an inhibitor against VEGF, vascular proliferation, and an anti-angiogenic molecule, rifampicin could be administered as an intravitreal injection, but without differentiating characteristics from current intravitreal injections, it would not provide a clinical advantage. On the other hand, as an eyedrop formulation in conjunction with intravitreal anti-VEGF injections, this combination has the potential to reduce treatment burden by prolonging the interval between eye injections. It has the potential to be used as a companion drug to extended injection intervals of anti-VEGF therapies.

Sustained release devices and/or gene therapy have their specific side effects. Sustained release devices can migrate into the anterior chamber, requiring surgical removal [45]. Gene therapy with viral vectors has the potential to incite inflammation in the retina [46]. The port delivery device to deliver ranibizumab requires surgery, which has had complications such as vitreous hemorrhage [47]. In summary, these methods to prolong the duration of the anti-VEGF effect carry different risks and may not alleviate the treatment burden to the patient and the health care system as envisioned.

Eyedrops remain the safest delivery method, but attempts at developing eyedrops containing TKIs for exudative AMD have been unsuccessful [17,48]. Rifampicin has several advantages over previously tested eyedrop therapeutics. Rifampicin is not a TKI, and the safety profiles of the compound are strong compared with those of TKIs [37,38]. However, it inhibits genes required for neovascularization, including VEGF, VEGF receptors (Flt-1 and KDR), the platelet/endothelial cell adhesion molecule 1, focal adhesion kinase, endothelin 1, endothelin receptor type B, integrin aV, integrin-b3, and c-myc, all in a dose-dependent fashion [29]. The inhibition of the various neovascularization genes may explain the significant discrepancy in the effective concentrations in vitro [30] and in vivo (Figure 5 and Figure 6).

The multifaceted MOA involved in inhibiting neovascularization by rifampicin could potentially offer enhanced effectiveness compared to earlier eyedrop formulations of TKIs. To date, all TKI eye drops have primarily targeted the VEGF pathway, showing no additional clinical benefits beyond those provided by anti-VEGF injections alone. The combined targeting of integrin and other vascular endothelial factors involved in neovascularization and vascular permeability, along with anti-VEGF properties, may confer an efficacy advantage to rifampicin over TKIs. This could allow the use of lower drug concentrations to achieve significant anti-angiogenic effects in the posterior segment of the eye.

Self-administration of eye drops potentially exhibits considerable variability in dosage and patient adherence [7]. Factors including manual dexterity, blink reflex, cognitive abilities, and overall compliance influence the quantity of medication that successfully contacts the eye’s surface and permeates to the posterior segment. The use of topical ointments, such as rifampicin ointments with less or no irritation, may mitigate some of these challenges. Moreover, in pre-clinical pharmacokinetic studies, the ointment formulations efficiently delivered approximately 10-fold the effective dose to the posterior eye segment. This efficient delivery could address the fluctuations commonly associated with topical applications. In the case of maintenance therapy as a companion drug to prolong injection intervals of anti-VEGF therapies, the challenges will be further mitigated because the occasional intravitreal injections will establish a solid baseline in the course of the treatment.

Although some injections and drug-eluting TKI implants [49,50], high-dose afliberecept [51], brolucizumab [52], and faricimab [53] can extend treatment intervals, they may inhibit VEGF more intensely, potentially leading to treatment-related complications in the back of the eye. Fibrosis and atrophy have been observed in the back of the eye following prolonged administration of repeated anti-VEGF injections over several years [54,55]. The correlation between these complications and the anti-VEGF therapies warrants further investigation. However, in general, highly intensive treatments may lead to complications that could compromise long-term vision preservation. To maximize visual acuity retention, patients should receive less invasive treatments, such as topical applications, and visit their ophthalmologists frequently. Depending on disease status and progression, ophthalmologists might adjust the dosing frequency of a topical product to minimize the potential complications. These treatment outcomes and benefits will also be achieved in both the monotherapies and maintenance therapies by the topical products to prolong the injection intervals.

The results presented in this report suggest the therapeutic potential of rifampicin eye drops for the treatment of exudative AMD. A regulatory pathway for drug repurposing will facilitate clinical trials of rifampicin eye drops due to their prior human use. This will enable initial exploratory clinical trials to enroll patients. These clinical plans will be validated in a pre-IND meeting with FDA officials. The successful clinical development of rifampicin eye drops could offer new treatment options for patients, addressing a critical gap, as no topical products are currently approved for exudative AMD. Furthermore, the use of rifampicin eye drops as monotherapy or in combination with existing anti-VEGF therapies to reduce the frequency of intravitreal injections could significantly enhance patients’ quality of life and contribute to long-term vision preservation.

## 4. Materials and Methods

### 4.1. 0.25% and 0.5% Rifampicin Water-Based Solubilized Formulations

Rifampicin was completely dissolved at the final concentrations of 0.25% and 0.5% at room temperature in the following eye drop formulations. The 0.25% rifampicin formulation: 10 mM Boric acid/Borax buffer (final pH 7.64), 150 mM NaCl, 0.5% Tween 80, 0.1% EDTA, 0.01% Benzalkonium chroride, and 0.25% rifampicin. The 0.5% rifampicin formulation: 18 mM Boric acid/Borax buffer (final pH 8.01), 0.9% NaCl, 0.5% Tween 80, 0.1% EDTA, 0.01% Benzalkonium chroride, and 0.5% rifampicin. Details are described in US patent No. 11,850,213 B2. Rifampicin was not precipitated in the formulation for several weeks. Rifampicin solutions were preserved at room temperature or in a refrigerator.

### 4.2. Topical Application of Rifampicin Reaches the Retina in Rat

Four male Sprague-Dawley rats (250 to 300 g) were used to measure the retina exposure level of rifampicin after the application of eye drops. Two rats received three drops of the eye drop formulation (0.25% rifampicin) in each eye under isoflurane sedation. A single drop contained 5 μL of the above-described formulation, and the application of each drug drop was carried out at intervals of 30 min. After the eye drop application, euthanasia was performed using CO_2_ gas, and the retina was excised from each rat under a dissection microscope. In addition, “non-treated” negative controls were used, and the retina was excised from two rats without performing any treatments on the rats. The retina was placed in a 1.5 mL microcentrifuge tube (1 retina/tube) and was fully washed with DPBS. After completion of the washing procedures, the retinal tissues in the microcentrifuge tube were frozen in dry ice and then preserved for quantification by LC/MS analysis.

### 4.3. Pharmacokinetic Studies Using a 0.25% Rifampicin Eye Drop Formulation

Six male Sprague–Dawley rats (250–300 g) were used to measure the retina exposure level of rifampicin after the application of the topical eye drops. The 0.25% rifampicin eye drop formulation (15 μL) was used, and the compound was administered to a single eye of each rat under isoflurane sedation. At time points of 1 h, 3 h, and 7 h after the application of the eye drops, euthanasia was performed using CO_2_ gas, and the retinal tissues were excised from the rats under a dissection microscope. In addition, “non-treated” negative controls were used, and the retina was excised from one rat without performing any treatments on the rat. The retina was placed in a 1.5-mL microcentrifuge tube (1 retina/tube) and was fully washed with DPBS. After completion of the washing procedures, the retinal tissues in the microcentrifuge tube were frozen in dry ice and then preserved for quantification by LC/MS analysis.

### 4.4. Preclinical Efficacy

Oxygen-induced retinopathy rat models were produced according to the protocols of Yanni et al. (2010) [56]. Sprague–Dawley rat babies (and their nursing mothers) were exposed to a cycling oxygen environment (80% and 21%, about one day for each) for 15 days after starting from the day of birth (Day 0). On Day 15 (P15), the animals were moved to room air. Six babies, seven babies, and six babies were assigned to the administration groups, namely a control group of only vehicles, a rifampicin eye drop formulation administration group, and an SC injection administration group, respectively. The 0.25% rifampicin eye drop formulation or a vehicle only was administered to the eyes of the baby rats every day for five days between P15 and P19, in the morning, at noon, and in the evening. The 0.5% rifampicin formulation was administered to the baby rats by SC injection at a dose of 20 mg/kg once a day from P15 to P19. On P20, all of the animals were euthanized by CO_2_ gas, and the retina was visualized as histology sections. In those histology sections, capillaries in the retinal tissues in three eyeballs (rifampicin topical application, rifampicin SC injection administration group, and non-induction of retinopathy) or five eyeballs (control administration of a vehicle only) were counted, so that neovascularization was quantified.

### 4.5. Determination of Effective Dose of Rifampicin in Mouse Laser-Induced CNV Models

The eyes of C57BL/6J mice were laser irradiated, and CNV was induced in sub-retina tissues in the eyes. Vehicle-only negative control or various concentrations of rifampicin dissolved in a water-based formulation were administered once daily by SC injections. Concurrent with the laser treatment, 2 μL of afliberecept (40 mg/mL) was intravitreally injected. Seven days after the laser irradiation, a FITC-dextran solution was administered to the tail vein. The eyeballs were removed under euthanasia by cervical dislocation, and the removed eyeballs were fixed with 4% paraformaldehyde phosphate buffer. The cornea, iris, and lens were excised, and retinal tissues other than retinal pigment epithelium cells were stripped using a microspacer. The optic cups were divided into four to six sections using a corneal microscissor, and they were placed on a slide glass. A confocal laser scanning microscope was used to photograph these fluorescent images that showed neovascularization induced in the sub-retina. The CNV areas were quantified using ImageJ (an image software publicly available by NIH). Pixels that were derived from fluorescent CNV areas in the eyes of the vehicle-only negative control were evaluated as 100%, and percentages of fluorescent pixels from dosing groups administered by various concentrations of rifampicin were evaluated. P-values against negative control results obtained from the vehicle-only dosing group were evaluated by the Williams multiple comparison test.

To evaluate the effective area under the curve (AUC) of rifampicin delivered to the mouse sub-retina, rifampicin was administered to C57BL/6J mice by SC injection at 0.7 mg/kg. This effective dosage and administration route was evaluated in dose escalation studies by using the mouse laser-induced CNV model. The AUC reflects the exposure to the drug after administration of a dose of the drug and is expressed in hour · ng/mg tissue. The AUC is dependent on the dose administered as well as the rate of elimination of the drug from the tissue. The AUC is directly proportional to the dose when the drug follows linear kinetics, and AUC is inversely proportional to the clearance of the drug. In 1 h, 3 h, 6 h, and 18 h after the SC injection, eyeballs were extracted. Tissue from the retina, the sclera, and the sub-retina complex were isolated and extracted. These tissues were immediately frozen in liquid nitrogen. The tissue was subjected to LC/MS analysis for quantitation. The detection limit was 0.25 ng/g tissue.

### 4.6. Oil- or Water-Based Formulations

Oil-based formulations (A–F; Table 6) comprising petrolatum and liquid paraffin mixed at various ratios were prepared, comprising sesame oil and light liquid paraffin. These components were mixed at room temperature. Rifampicin was formulated therein at 1% (*w*/*w*). Table 6 depicts the measured viscosities of the oil-based formulations (A–F) that were measured at 25 °C with a shear velocity of 200 s^−1^ using a Modular Compact Rheometer (MCR302, Anton Paar). A temperature control unit (P-PTD200) and a shaft (CP25-2) were used in these measurements. The temperature for each measurement was controlled and maintained at least in the range of 25.0 ± 0.1 °C.

Water-based suspension formulations (G and H; Table 7) were prepared with NaCl in the presence or absence of a viscosity-impairing agent (cellulose polymers), respectively. A water-based solubilized formulation (RK32) was prepared with polyoxyethylene castor oil, ethylene glycol monostearate, and a viscosity-imparting agent (cellulose polymers). Rifampicin was formulated therein at 1% (*w*/*w*). All components were mixed at room temperature. The pH of the formulations H and RK32 was adjusted to about 7.0 and 8.4 by using phosphate buffer, respectively. The pH of formulation G was adjusted to about 7.0 by using phosphate buffer. Table 7 depicts the measured viscosities of the water-based formulations (G, H, and RK32) at 25 °C with a shear velocity of 200^−1^ by using a Modular Compact Rheometer (MCR302, Anton Paar). In these measurements, a temperature control unit (P-PTD200) and a shaft (CP25-2) were used. During the measurements, the temperature was controlled and maintained at least in the range of 25.0 ± 0.1 °C.

The relationship between viscosity and shear velocity was determined for oil-based formulations (A–F) and water-based formulations (G, H, and RK32) at 25 °C. Shear is the relative motion between adjacent layers of a fluid. Shear velocity is the rate of change of velocity at which a fluid layer passes over another adjacent fluid layer. The relationship between viscosity (mPaS) and shear velocity (s^−1^) for each formulation is described in PCT patent publication No. WO 2023/234423 A1.

Oil-based formulations (C and E; Table 8) comprising petrolatum and liquid paraffin were prepared, and rifampicin was formulated therein at 0.01% (*w*/*w*) and 0.001% (*w*/*w*). Table 8 describes the measured viscosities of the oil-based formulations that were measured at 25 °C with a shear velocity of 200^−1^ by using a Modular Compact Rheometer (MCR302, Anton Paar). A temperature control unit (P-PTD200) and a shaft (CP25-2) were used in these measurements. During the measurements, the temperature was controlled and maintained at least in the range of 25.0 ± 0.1 °C.

The purity of rifampicin and its related compounds, including degradation products, was confirmed using HPLC methods essentially in accordance with the procedures described in the Japanese Pharmacopoeia [57].

### 4.7. Ocular Tissue Delivery of Rifampicin via Oil-Based, Water-Based Suspension, and Water-Solubilized Formulations by Topical Applications

Rabbits (Kbs:JW) were placed under anesthesia by injecting a mixture of ketamine/xylazine. Both 20 μL of oil- and water-based formulations (Table 6, Table 7 and Table 8, A–H) and 50 μL of a water-based formulation (RK32) contained 1% rifampicin. The formulations were topically dosed to the eyes of the rabbits. One hour after the topical applications of the formulations (A–F and H), blood samples were collected from the rabbits, and plasma fractions of the blood samples were saved in vials. In 1 h and 18 h after the topical applications of the formulations (A–F), the rabbits were euthanized by intravenous injection of sodium pentobarbital, and eyeballs that were dosed in the formulations were extracted. Eyeballs were dissected, and vitreous, retina, sub-retina, and scleral tissues were prepared and immediately frozen in liquid nitrogen. In 0.5 h, 1 h, 3 h, and 6 h after the topical application of the water-solubilized formulation RK32; in 1 h, 3 h, 6 h, and 18 h after the topical applications of the oil-based and water-based suspension formulations (B, C, F, and H); and in 1 h and 6 h after the topical application of the water-based suspension formulation (G), plasma fractions of blood samples and eyeball tissues, including the retina and sub-retina tissues, were collected and extracted. They were immediately frozen in liquid nitrogen. These eye tissues and plasma samples were subject to LC/MS analysis for quantification. The detection limit was 0.25 ng/g tissue.

### 4.8. Oil-Based Formulation Plasma Exposure

In the pharmacokinetic studies described in Section 2.7, oil-based formulations (A–D) containing 1% rifampicin were topically applied to the eyes of rabbits. To evaluate the systemic exposure to rifampicin, blood samples were collected from the rabbits 1 h after the topical application, and plasma fractions were prepared from the blood samples. Rifampicin amounts in the plasma fractions were quantified by liquid chromatography-mass spectroscopy. The detection limit was 0.25 ng/mL plasma.

## 5. Conclusions

The development of rifampicin eyedrop formulations for the treatment of retinal diseases of abnormal neovascularization is expected to serve as an adjunctive therapy to intravitreally injected therapeutics, or these formulations will potentially eliminate the need for intravitreal injections. The rifampicin formulations will alleviate the treatment burden of frequent eye injections.

## 6. Patents and Patent Applications

The following patents and patent applications resulted from this work:

US 11,850,213 B2

US 10,709,702 B2

US Patent Application No. 18/267,715

WO 2023/234423 A1

## Figures and Tables

**Table 1:** Drug amount detected in the retina compared to the drug amount administered for various compounds.

Compound (Brand Name) Mechanism of Action for Indication or Usage	Drug Amount Administered	Drug Amount Detected in Retina
Betoptic 0.5% (MW: 307) Beta 1 receptor blocker for glaucoma [39]	0.5% 4 drops (120–200 μL, 600–1000 μg)	300 ng/ 1 g of retinal tissues (Rabbit, average of n = 5)
	0.5% 8 drops (240–400 μL, 1200–2000 μg)	480 ng/ 1 g of retinal tissues (Rabbit, average of n = 7)
Dexamethasone (MW: 392) Glucocorticoid for inflammation [40]	0.5% 50 μL, 250 μg	33 ng/ 1 g of retinal tissues (Rabbit, average of n = 6)
Rifampicin	0.25% 3 drops (15 μL, 37.5 μg)	1280 ng/ 1 g of retinal tissues (Rat) SE: 394.0348
	0.25% 10 drops (50 μL, 125 μg)	2939 ng/ 1 g of retinal tissues (Rat) SE: 50.54041

**Table 2:** Sub-retina.

	(n = 3, Average) Mouse effective AUC (h·ng/mg tissue)	AUC by oil- based formulation A (h·ng/mg tissue)	AUC by oil- based formulation B (h·ng/mg tissue)	AUC by oil- based formulation C (h·ng/mg tissue)	AUC by oil- based formulation D (h·ng/mg tissue)
AUC values	0.27	0.96	1.37–2.11	2.18–2.68	4.40
Folds Greater Than Effective AUC	X1	X4	X5–X8	X8–X10	X16
	AUC by oil- based formulation E (h·ng/mg tissue)	AUC by oil-based suspension formulation F (h·ng/mg tissue)	AUC by water-based suspension formulation G (h·ng/mg tissue)	AUC by water-based solubilized formulation H (h·ng/mg tissue)	AUC by water-based solubilized formulation RK32 (h·ng/mg tissue)
AUC values	8.77	0.62	1.82	1.33	2.05
Folds Greater Than Effective AUC	X32	X2	X7	X5	X8

**Table 3:** Retina.

	(n = 3, Average) Mouse effective AUC (h·ng/mg tissue)	AUC by oil- based formulation A (h·ng/mg tissue)	AUC by oil- based formulation B (h·ng/mg tissue)	AUC by oil- based formulation C (h·ng/mg tissue)	AUC by oil- based formulation D (h·ng/mg tissue)
AUC values	0.27	0.27	0.31–0.46	0.74	1.31
Folds Greater Than Effective AUC	X1	X1	X1–X2	X3	X5
	AUC by oil- based formulation E (h·ng/mg tissue)	AUC by oil- based formulation F (h·ng/mg tissue)	AUC by water-based suspension formulation G (h·ng/mg tissue)	AUC by water-based suspension formulation H (h·ng/mg tissue)	AUC by water-based solubilized formulation RK32 (h·ng/mg tissue)
AUC values	1.06	0.17	0.52	0.46	0.22
Folds Greater Than Effective AUC	X4	X1	X2	X2	X1

**Table 4:** Plasma: Rifampicin concentrations (ng/mL plasma) in 1 h after the topical dosing.

	A	B	C	D	RK32
Rifampicin (ng/mL plasma)	0.70	0.47	1.59	1.96	10.24
SE	0.2144	0.0982	0.2764	0.3129	0.9431

Average n = 3.

**Table 6:** Oil-based formulations containing rifampicin.

	A	B	C	D	E	F
Rifampicin	1%	1%	1%	1%	1%	1%
White Petroleum	99%	79.2%	49.5%			
Liquid Paraffin		19.8%	49.5%	99%		
Sesame Oil					99%	
Light Liquid Paraffin						99%
Viscosity (mPaS)	3815	2145	867	161	63	6
Shear Velocity (s^−1^)	200	200	200	200	200	200

**Table 7:** Water suspension and water-solubilized formulation containing rifampicin.

	G	H	RK32
Rifampicin	1%	1%	1%
NaCl	0.67%	0.4%	
Polyoxyethylene castor oil			5%
Ethylene glycol monostearate			3%
Cellulose polymers	0.8%		0.5%
Na_2_HPO_4_	0.3%	1.5%	1.5%
EDTA		0.1%	0.1%
Anti-oxidants		0.3%	0.3%
pH	7.1	7.0	8.3
Viscosity (mPaS)	101	1.3	39.7
Shear Velocity (s^−1^)	200	200	200

**Table 8:** Oil-based formulations containing various concentrations of rifampicin.

	C (0.01% Rifampicin)	C (0.001% Rifampicin)	E (0.1% Rifampicin)	E (0.01% Rifampicin)
Viscosity (mPaS)	801	781	62	65
Shear Velocity (s^−1^)	200	200	200	200

## Data Availability

The datasets presented in this article are not readily available because they are owned by AMD Therapeutics and its co-development partners. Requests to access the datasets should be directed to Hiroaki Serizawa.

## Supplementary Materials

The following supporting information can be downloaded at https://www.mdpi.com/article/10.3390/ph18050655/s1, Figure S1: Representative images of the histology sections.
