# Supplementary material for: Development of Rifampicin Eye Drops for the Treatment of Exudative Age-Related Macular Degeneration
Source: Pharmaceuticals (Basel). 2025 Apr 29;18(5):655. doi: 10.3390/ph18050655 (PMC12115180; doi:10.3390/ph18050655)
Supplement: Supplementary file 1 [file pharmaceuticals-18-00655-s001.zip › pharmaceuticals-3552106-Figure S1.pdf]

Hyperoxygenated with vehicle only treatment

There are increased numbers of small new capillaries on the retinal surface. 200x, HE

There are increased numbers of small new capillaries on the retinal surface. 400x

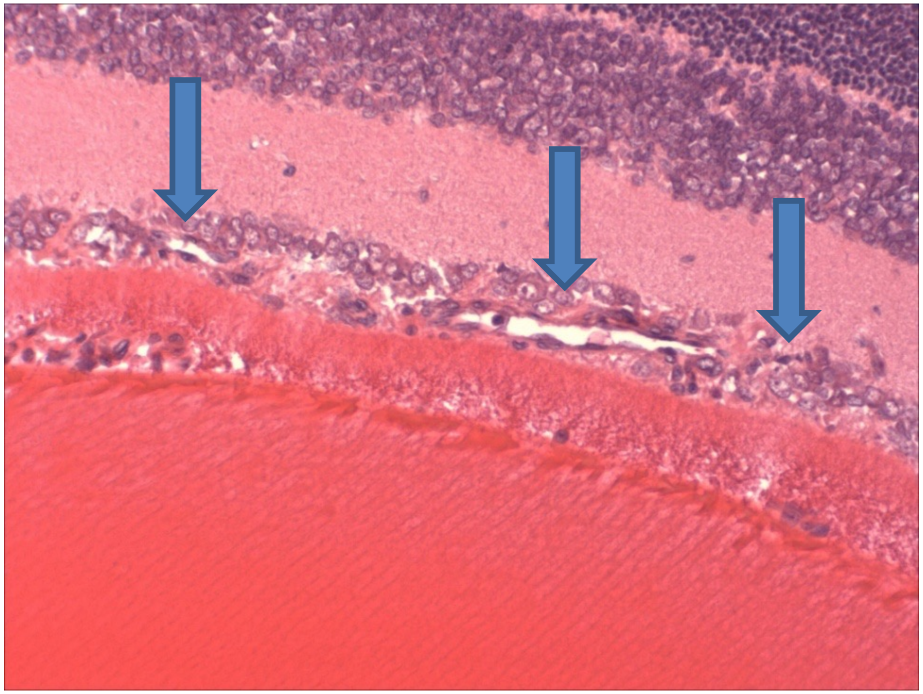

A

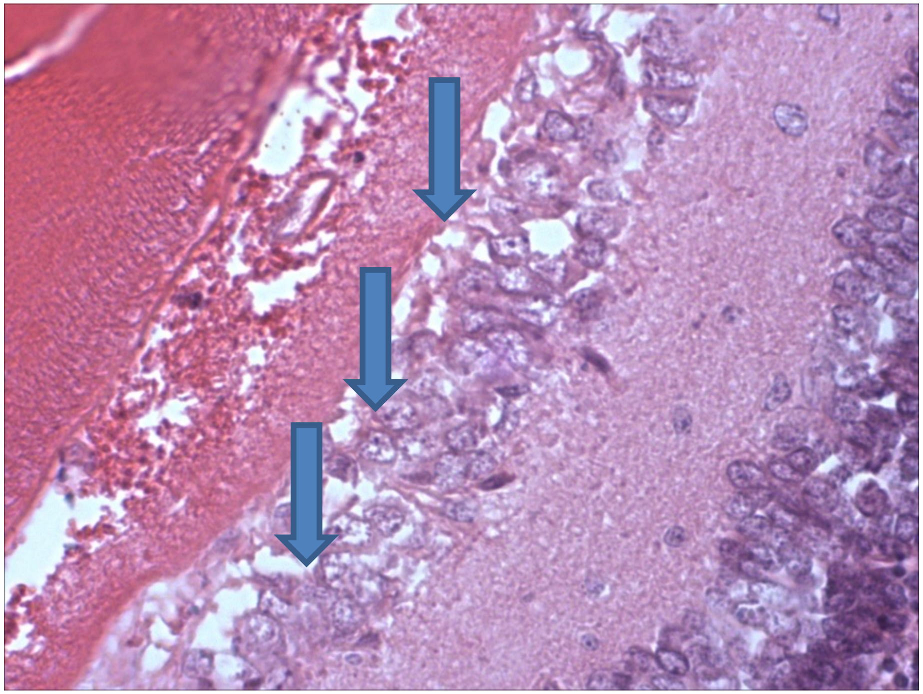

B

Rifampicin topical

There are small foci of new capillaries on the retinal surface, but fewer than in the untreated Group 1. 200x, HE

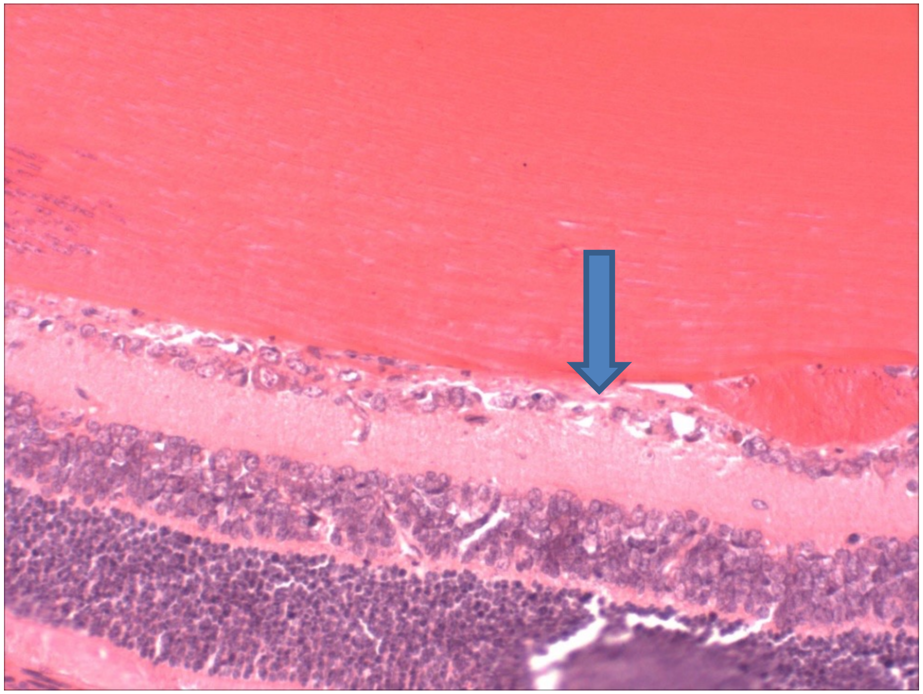

C

There are small foci of new capillaries on the retinal surface, but fewer than in the untreated Group 1. 400x, HE

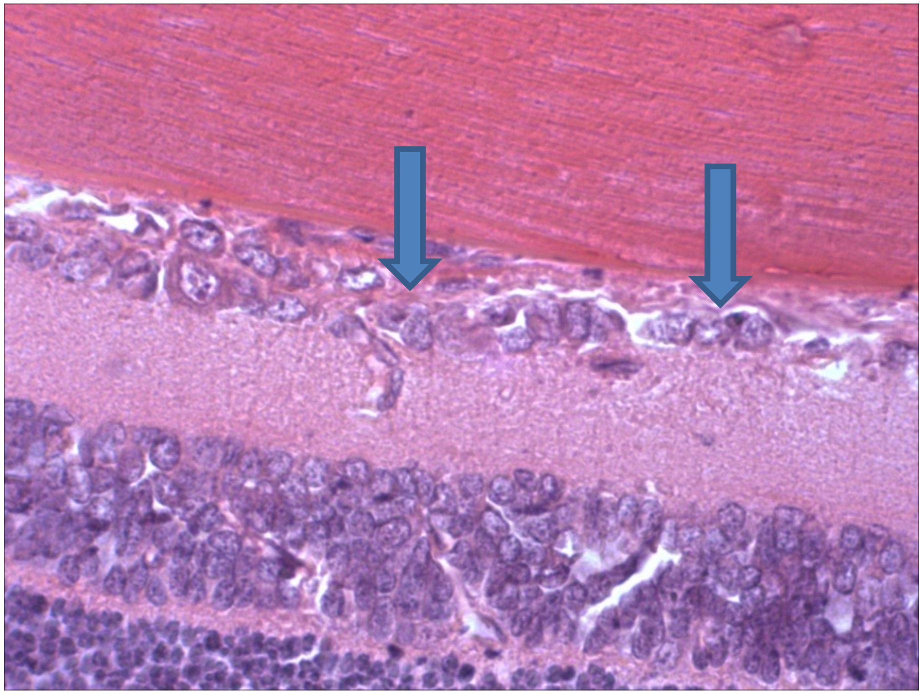

D

Rifampicin subcutaneous

There are small foci of new capillaries on the retinal surface, but fewer than in the untreated Group 1. 200x, HE

There are rare small new capillaries on the retinal surface, fewer than in the untreated Group 1. 400x, HE

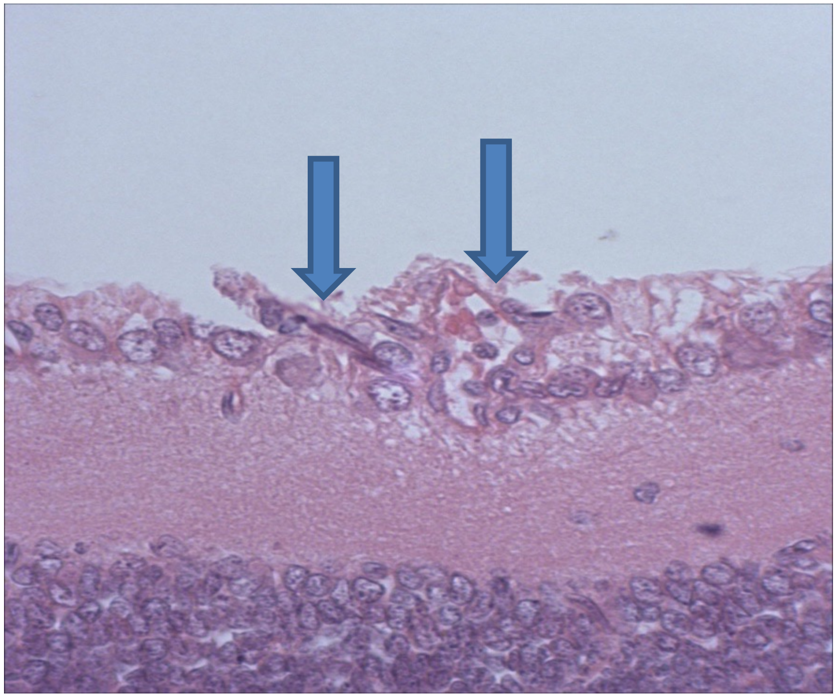

E

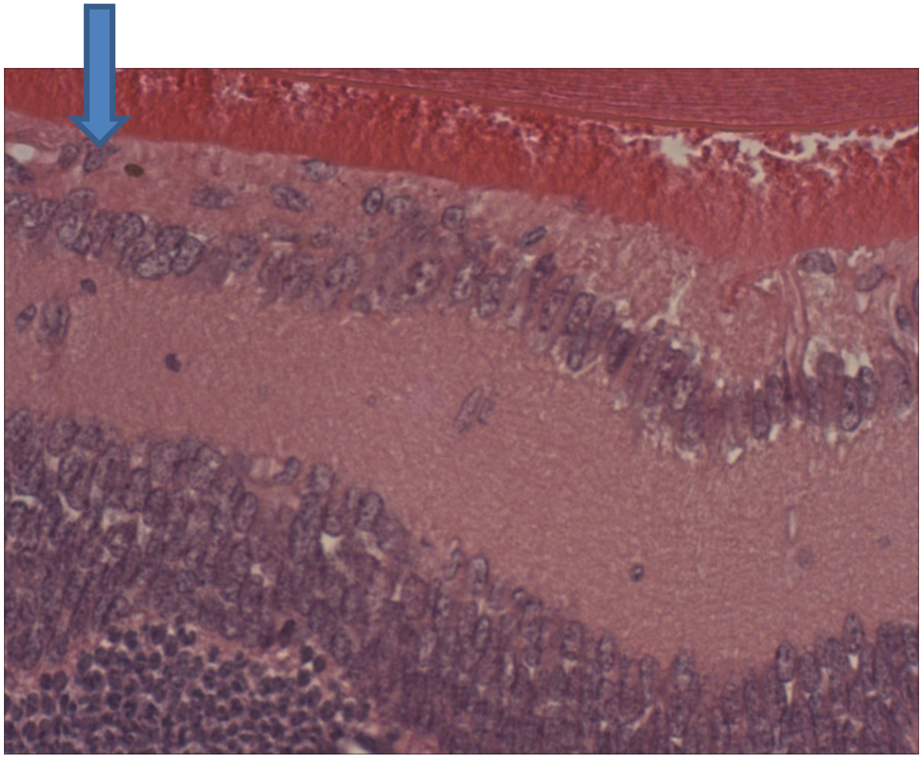

F

Normal eyes

There are a few small vessel cross sections visible, but no new capillaries. 200x HE

There are no small vessel cross sections visible, and no new capillaries. 400x HE

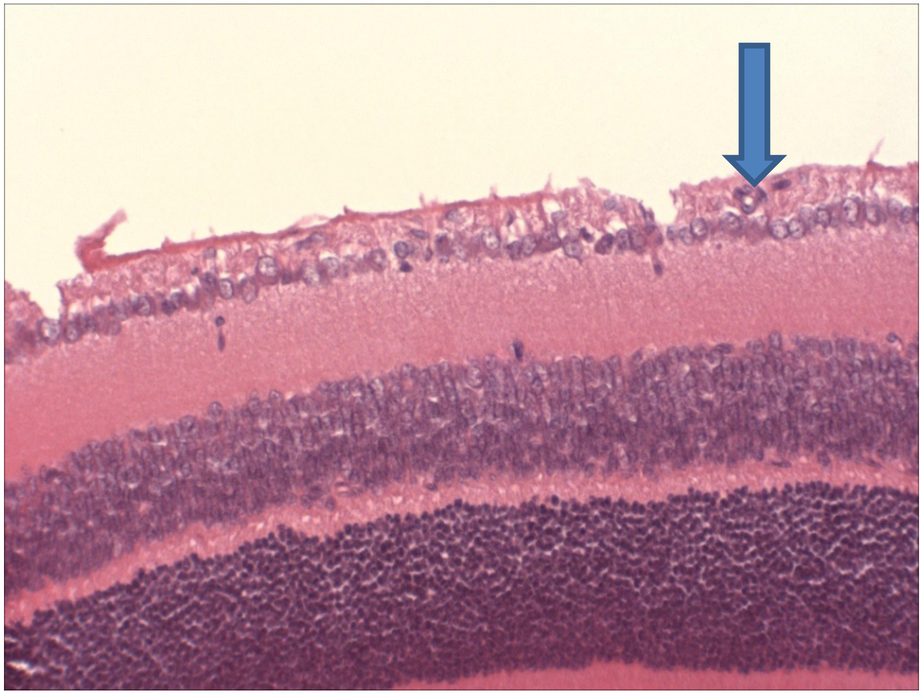

G

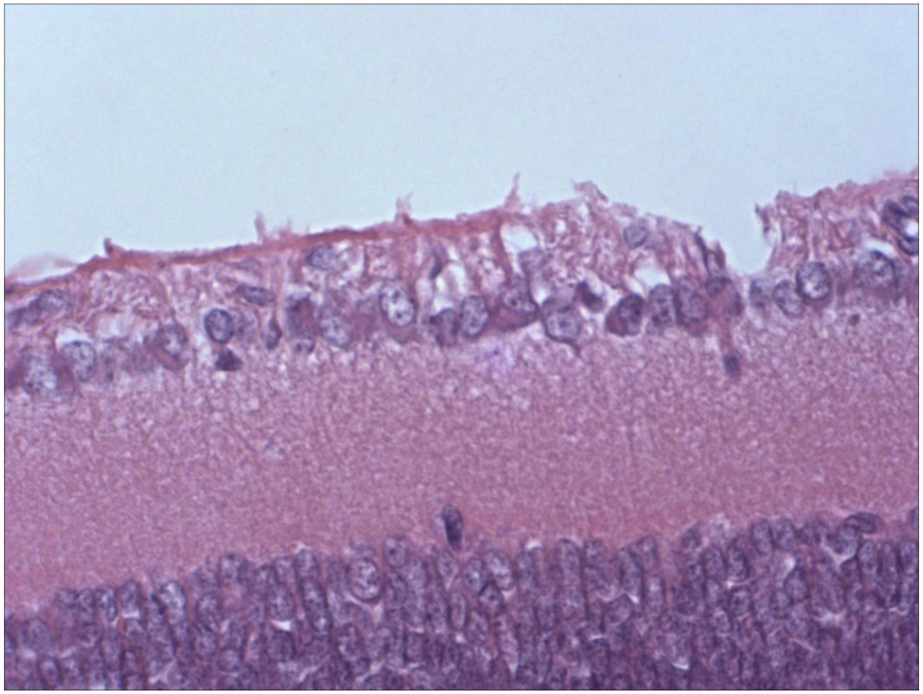

H
